# New Insight into Adiponectin Role in Obesity and Obesity-Related Diseases

**DOI:** 10.1155/2014/658913

**Published:** 2014-07-07

**Authors:** Ersilia Nigro, Olga Scudiero, Maria Ludovica Monaco, Alessia Palmieri, Gennaro Mazzarella, Ciro Costagliola, Andrea Bianco, Aurora Daniele

**Affiliations:** ^1^CEINGE-Biotecnologie Avanzate Scarl, Via Salvatore 486, 80145 Napoli, Italy; ^2^Dipartimento di Medicina Molecolare e Biotecnologie Mediche, Università degli Studi di Napoli Federico II, Via De Amicis 95, 80131 Napoli, Italy; ^3^Dipartimento di Scienze Cardio-Toraciche e Respiratorie, Seconda Università degli Studi di Napoli, Via Bianchi 1, 80131 Napoli, Italy; ^4^Cattedra di Malattie dell'Apparato Visivo, Dipartimento di Medicina e Scienze della Salute, Università del Molise, Via De Sanctis 1, 86100 Campobasso, Italy; ^5^Cattedra di Malattie dell'Apparato Respiratorio, Dipartimento di Medicina e Scienze della Salute, Università del Molise, Via De Sanctis 1, 86100 Campobasso, Italy; ^6^Dipartimento di Scienze e Tecnologie Ambientali Biologiche Farmaceutiche, Seconda Università degli Studi di Napoli, Via Vivaldi 42, 81100 Caserta, Italy

## Abstract

Obesity is a major health problem strongly increasing the risk for various severe related complications such as metabolic syndrome, cardiovascular diseases, respiratory disorders, diabetic retinopathy, and cancer. Adipose tissue is an endocrine organ that produces biologically active molecules defined “adipocytokines,” protein hormones with pleiotropic functions involved in the regulation of energy metabolism as well as in appetite, insulin sensitivity, inflammation, atherosclerosis, cell proliferation, and so forth. In obesity, fat accumulation causes dysregulation of adipokine production that strongly contributes to the onset of obesity-related diseases. Several advances have been made in the treatment and prevention of obesity but current medical therapies are often unsuccessful even in compliant patients. Among the adipokines, adiponectin shows protective activity in various processes such as energy metabolism, inflammation, and cell proliferation. In this review, we will focus on the current knowledge regarding the protective properties of adiponectin and its receptors, AdipoRs (“adiponectin system”), on metabolic complications in obesity and obesity-related diseases. Adiponectin, exhibiting antihyperglycemic, antiatherogenic, and anti-inflammatory properties, could have important clinical benefits in terms of development of therapies for the prevention and/or for the treatment of obesity and obesity-related diseases.

## 1. Introduction

Obesity is due to excessive fat accumulation that may impair health resulting from social behaviour and environmental and genetic factors [1, 2]. During the last 20 years, obesity has rapidly become a global pandemic health problem: catastrophic data come from America and from Europe where ~35% and ~20% of the population, respectively, are obese [3–5]. Globally, the World Health Organization (WHO) has predicted that, in 2015, ~2,3 billion of adults will be overweight; 700 million will be obese, while ~200 million of school aged children will be obese/overweight (http://www.IASO.org/). The major risk factors for developing obesity are environmental and genetic. In 1962, J. Neel theorized the “thrifty gene hypothesis” to partially explain the rise in obesity-related diseases in the world [6]. According to this, various genes promoting the efficient utilization and storage of fuel would have been favored by natural selection to allow the survival of the human race during famines while today, in times of food abundance, they predispose to obesity and type 2 diabetes mellitus (T2DM) [6]. Genetic alterations predispose to obesity by increasing the risk of disease development by 40–70% [1, 7]. So far, more than 200 candidates' genes in mice and more than 100 in humans have been implicated in body weight regulation [7, 8]. In particular, the genes responsible for the monogenic form of obesity are leptin, leptin receptor, melanocortin receptor 4, proopiomelanocortin, prohormone convertase 1, and Agouti related protein [9–12]. In addition, the genes having high scores of association with obesity are fat mass and obesity associated, catenin *β*-like 1, v-maf musculoaponeurotic fibrosarcoma oncogene 7 homolog, transmembrane protein 18, phosphodiesterase related, Niemann-Pick disease type C1, prolactin, and obesity candidate gene G protein *β*3 [12–14]. Genome-wide association studies identified a number of loci correlated to adult and childhood obesity [15–17]. Moreover, recently, large deletions and duplications that represent copy number variation (CNV) have been linked to the early onset obesity in children [13]. The complexity of the “obesity problem” has become clearer since adipose tissues have been recognized as an endocrine organ that produces biologically active substances defined as “adipokines,” protein hormones with pleiotropic functions in the regulation of energy metabolism as well as in appetite, insulin sensitivity, inflammation, atherosclerosis, and proliferation [18, 19]. Amongst the others, the most biological relevant adipokines are leptin, plasminogen activator inhibitor (PAI-1), tumor necrosis factor (TNF-*α*), interleukin 6 (IL-6), resistin, and adiponectin [19, 20]. The latter plays a pivotal role in various processes such as energy metabolism, inflammation, and cell proliferation. In this review, we will focus on insulin sensitizing, antiatherogenic, and anti-inflammatory properties of adiponectin and its AdipoRs. We will describe the latest knowledge on the role of adiponectin and its AdipoRs in obesity and obesity-related diseases.

## 2. Adiponectin

### 2.1. Biology

Adiponectin, also known as adipocyte complement-related protein of 30 kDa (Acrp30), was identified by different groups [21–24]. Adiponectin is an adipokine abundantly produced and secreted by adipose tissues and widely recognized for its antidiabetic, anti-inflammatory, antiatherogenic, and cardioprotective effects [25–27]. Adiponectin is a protein hormone of 244 amino acids that circulates in high concentrations (5–30 *μ*g/mL) accounting for 0.01% of total serum proteins. Adiponectin expression and serum levels are decreased in obese patients, pigs, and rodents [28, 29]. Sexual dimorphism has been observed in adiponectin expression, with males showing lower levels than females [28]. Adiponectin is synthesized as a monomer of 28–30 kDa that is assembled in homooligomers of various molecular weights: low molecular weight (LMW) trimeric form, medium molecular weight hexameric (MMW), and high molecular weight (HMW) [24, 30]. The monomeric peptide sequence is composed of four regions: an amino-terminal peptide, a short hypervariable region, a collagen-like domain containing 22 Gly-X-Pro or Gly-X-Y repeats, and a carboxy-terminal globular domain C1q like [24]. In serum, the adiponectin monomeric form is present as a full-length form (*f*Adiponectin) or as a globular form of the protein (*g*Adiponectin) [31, 32].* g*Adiponectin is generated through proteolytic cleavage product of* f*Adiponectin and contains the globular head without the collagen-like domain enabling the formation of trimers but not HMW oligomers [33, 34]. Recently, it has been observed that the monomeric form stimulates AMPK activation in muscle and increases fatty acid oxidation and peripheral glucose uptake [35]. Furthermore, an increase of the* g*Adiponectin has been correlated to the improvement of whole-body energy metabolism and of adipose tissue functions [29, 36]. Adiponectin undergoes extensive and complex posttranslational processing critical to the formation and secretion of adiponectin multimers; in humans, the most important modification is a hydroxylation and glycosylation of four conserved lysine residues (lys65, lys68, lys77, and lys101) and/or the hydroxylation of proline residues within the collagenous domain; assembly of human adiponectin oligomers depends on the disulfide bond formation mainly mediated by cysteine 39 [37]. Furthermore, different adiponectin complexes do not interconvert after secretion [38]. Adiponectin homooligomers assembly of different molecular weights is an important process strongly correlated to the biological functions of this adipokine. It is well known that HMW oligomers are the major relevant forms in insulin sensitivity activities of adiponectin and that low amounts of HMW oligomers represent an independent risk factor for metabolic pathologies such as obesity-related diseases [39]. For these reasons, HMW/total adiponectin ratio seem to be more useful than total adiponectin for the assessment of the risk of several diseases including obesity, insulin resistance (IR),T2DM, metabolic syndrome (MetS), and cardiovascular diseases (CVDs) [29, 40–42].

Adiponectin is encoded by APM1 gene (adipose most abundant gene transcript1) composed of three exons spanning 16 kb. APM1 is localized on the long arm of chromosome 3 in the 3q27 region reported as closely associated with various quantitative trait loci (QTL) for the MetS and T2DM [43]. Some rare mutations and specific single-nucleotide polymorphisms (SNPs) have been identified in APM1 gene; the formers are significantly related to T2DM and hypoadiponectinemia while the studies for the SNPs reported controversial results [43–49].

### 2.2. Adiponectin Receptors

Adiponectin acts through two major functionally distinct and ubiquitously expressed receptors, AdipoR1 and AdipoR2; the former is the most abundant form in skeletal muscle, whereas the latter is the most abundant form in the liver [24]. In addition, AdipoR1 has a very strong affinity for* g*Adiponectin whereas AdipoR2 has a very strong affinity for* f*Adiponectin [24]. AdipoR1 and AdipoR2 are seven transmembrane G-protein coupled receptors (GPCRs) but, as members of the PAQR (progestin and AdipoQ receptor) family, the N-terminus end is found in the cytoplasmic region of the cell whereas the C-terminus end is found externally [24]. After adiponectin activation, AdipoR1 and AdipoR2 assemble in both homo- and heterodimeric complexes [24]. Both receptors have a physiological relevance in metabolic processes. Successively in 2004, T-cadherin, a member of the cadherin superfamily, was identified as a potent receptor for hexamers as well as HMW adiponectin oligomers [50]. In conclusion the adiponectin pathway depends on the molecular form of adiponectin, on the relative abundance of its receptors, and on the target tissue [51].

### 2.3. Adiponectin Actions in Energy Metabolism

Adiponectin exhibits key metabolic functions on skeletal muscle and liver [26]. In muscle, the insulin sensitivity functions of adiponectin are mediated via AMP kinase (AMPK) and peroxisome proliferator-activated receptor *α* (PPAR*α*) [24, 52]. In liver, adiponectin activates glucose transport and inhibits gluconeogenesis via AMPK, whereas adiponectin activates fatty acid oxidation and decreases inflammation through the PPAR*α* pathway [53]. Interestingly, activation of AMPK seems to be mediated mainly by AdipoR1, whereas activation of PPAR-*α* seems to be mediated by AdipoR2 [52]. In addition, adiponectin, in liver, enhances insulin sensitivity promoting phosphorylation of the insulin receptor and of the adaptor protein insulin receptor substrate 1 (IRS-1) [52]. In pancreas, adiponectin acts on cell proliferation stimulating insulin secretion [54]. In adipose tissues, adiponectin increases basal glucose uptake and enhances insulin-stimulated glucose uptake through AMPK activation [53]. In addition,* in vitro* studies demonstrated that adiponectin regulates fat lipid metabolism inhibiting lipolysis [55, 56]. However, a significantly increase of lipolysis is observed in both adiponectin gene knockout mice and primary adipocytes from these mice [55].

### 2.4. Adiponectin Actions in Inflammation

Adiponectin exhibits protective activity in several inflammatory diseases including atherosclerosis, CVDs, and IR [40, 57, 58]. Moreover,* in vitro* studies showed that adiponectin attenuates inflammation in endothelial, muscle, epithelial cells, and macrophages mainly by AMPK and cyclic AMP-protein kinase A (cAMP-PKA) activation [59, 60]. Molecular mechanisms of adiponectin may be direct actions on inflammatory cells suppressing reactive oxygen species and stimulating the expression of the anti-inflammatory IL-10 cytokine, suppression of the NF-*κ*B inflammatory signaling pathway, and downregulation of inflammatory responses involving TNF-*α* [58, 60–63].

### 2.5. Adiponectin Effects on Cell Proliferation

Recently,* in vitro* studies have demonstrated adiponectin involvement in various cancer cell types including breast, endometrial, colon, stomach, prostate, and leukemia [64, 65]; Adiponectin, in fact, inhibits cell growth and induces apoptosis in dose-dependent manner both* in vitro* and* in vivo* through different molecular pathways (for review, see [66, 67]). Through AMPK stimulation, adiponectin counteracts carcinogenesis by p21 and p53 that in turn regulate growth arrest and apoptosis in colon cancer cells [68, 69]. Moreover, tumor suppressor effects of adiponectin are mediated via AKT and ERK signaling pathways in lung and pancreatic cell lines [55, 60, 67]. However, a potential proliferative and antiapoptotic role of adiponectin has also been suggested by separate investigations [70, 71]. Epidemiologic studies have highlighted associations between decreased adiponectin levels with cancer development with some exceptions. In fact, in patients affected by breast and endometrial cancer, adiponectin levels are decreased; inversely, in patients affected by lung, prostate, gastric, liver, pancreatic and hematological, colon, and renal cancers adiponectin levels are increased.

## 3. Adiponectin and Obesity

Obesity is a major health problem increasing risk for MetS, CVDs, respiratory disorders, diabetic retinopathy, and cancer. In obese patients, visceral body fat may affect health conditions, through an abnormal production of adipokines. Adiponectin plays a pivotal role in energy metabolism; concentration of both total adiponectin and HMW decreases in obesity and increases after weight loss [28, 29, 72, 73]. In addition, total and HMW adiponectin oligomers are inversely correlated to BMI, glucose, insulin and triglyceride levels, degree of IR, and, importantly, visceral fat accumulation [29]. Numerous studies demonstrated a strong correlation between HMW and several metabolic abnormalities, while the role of MMW and to a lesser extent of LMW oligomers has been poorly investigated [3, 41, 74]. The manner by which adipose tissue expands (increases in size, hypertrophy, and/or in number of cells, hyperplasia) could regulate synthesis and secretion of adiponectin. Drolet et al. demonstrated an inverse relationship between mean adipocytes diameter and adiponectin secretion [75]. AdipoR1 and AdipoR2 expression is significantly decreased in T2DM and obesity state [75]. In obesity and T2DM, alterations in the expression of adiponectin and its AdipoRs reduce adiponectin sensitivity leading to IR which in turn aggravates hyperinsulinemia. After weight loss, adiponectin levels rise together with a specific increase of the most biologically active oligomers HMW. Interestingly, a recent work found that, in severe obesity (BMI ≥ 40 Kg/m^2^), following weight loss (about 10% weight), not only total and HMW adiponectin, but also the monomeric form levels increase [29]. This finding could suggest a functional recovery of adipose tissue after weight loss in severely obese patients.

Genetic alterations were correlated to adiponectin expression in metabolic diseases [76]; for example, p.G48R, P.Y111H, p.R112C, and p.G90S mutations are strongly associated with low levels of total adiponectin and HMW oligomers in patients affected by T2DM and obesity [43, 45, 46, 77]. Furthermore, 12 SNPs in adiponectin gene (APM1) were identified; some of these SNPs and haplotypes are correlated to hypoadiponectinemia, IR, and increased risk of T2DM [43]. In German and North America subjects, SNP +45 is associated with diabetes and IR; in French subjects, c.11377 and c.11391 SNPs are significantly associated with low levels of adiponectin and T2DM; in Italy, c.11377 SNP resulted to be correlated to adiponectin levels in severe obese patients [43, 47, 78]. Recently, haplotype analysis in 631 non-Hispanic white and 553 African-American subjects identified a strong link between noncontiguous ACDC haplotypes and adiponectin levels [79].

## 4. Adiponectin and Obesity-Related Diseases

The expansion of adipose tissues results in oxidative stress and inflammatory responses. Moreover, through a cross talk between adipocytes and the immune system, a significant infiltration in adipose tissue of immune and inflammatory cells is generated increasing local and successively systemic levels of various inflammatory cytokines. The dysregulation of cytokines and adipokines production strongly contributes to the onset of several obesity complications as MetS, CVDs, respiratory disorders, diabetic retinopathy, and cancer [80–82]. We will focus on the role of adiponectin.

### 4.1. Metabolic Syndrome

Metabolic syndrome (MetS), an emerging public health problem, is multiplex metabolic risk factors associated with a 5-fold risk of T2DM and a 2-fold risk of CVDs. Recently, chronic low-grade inflammation has been implicated among the major factors in the development of the MetS [83]. The inflammatory state in MetS is represented by elevated concentrations of a variety of inflammatory regulators such as C-reactive protein, TNF-*α*, resistin, IL-6, IL-8, visfatin, and adiponectin. The latter is inversely related to both adiposity and proinflammatory cytokines [84]. In addition, it was demonstrated that low HMW adiponectin levels are independently associated with the development of MetS [39]. Furthermore, lower adiponectin levels are found in adult patients, as well as in children with MetS [39]. Conversely, Kim et al. demonstrated that higher adiponectin levels are protective for incident metabolic syndrome in men and women and predict new-onset metabolic syndrome [85]. Recently, it has been demonstrated that, in patients with MetS compared to control subjects, skeletal muscle mRNA expression of AMPK*α* is lower, whereas the expression of AdipoR1 is upregulated [24]. On the other hand,* in vivo* studies on genetic overexpression studies or administration of recombinant adiponectin revealed positive metabolic effects [24]. In macrophages, adiponectin supplementation suppresses the production and secretion of proinflammatory cytokines TNF-*α* and IL-6, decreasing the synthesis of monocyte adhesion molecules in endothelial cells [58]. In parallel, adiponectin enhances the production of anti-inflammatory cytokines in epithelial cells and macrophages [60, 86].

### 4.2. Hypertension

The relationship between obesity and hypertension is well established and attributed to many factors among which are sympathetic nervous system activation, endothelial dysfunction (caused by increase in free fatty acids, oxidative stress), and an aberrant adipokine production [87]. In fact, lower adiponectin levels are detected in adults with hypertension [85]. Accordingly, total adiponectin levels have been found to be lower in obesity-associated hypertensives than in lean hypertensives or lean normotensives [88]. Iwashima et al. analyzed endothelial function in hypertensive patients, finding a positive correlation between serum adiponectin level and vasodilator response to reactive hyperemia [89]. They also found that, in adiponectin-KO mice, endothelial function is significantly reduced by inhibition of endothelial adhesion molecules as well as macrophage-to-foam cell transformation [89]. Several studies evidenced that adults with hypertension have lower adiponectin levels than normotensive adults and that the increase in adiponectin levels has been associated with reduced risk of hypertension. Adiponectin is a biologically relevant modulator of vascular remodeling linking obesity and vascular disease. Adiponectin protectively regulates blood pressure via brain- and endothelium-mediated mechanisms [89, 90]. In fact,* in vitro* studies have shown that adiponectin inhibits the expression and the biological effects of TNF*α*, of adhesion molecules, and the macrophage-to-foam cell transformation [91]. The antiatherogenic properties of adiponectin are mainly due to NO production in endothelial cells, using phosphatidylinositol 3-kinase-dependent pathways, as well as AMPK pathway [89, 90]. The NO production, in physiologic condition, relaxes vessels and exerts anti-inflammation and antithrombotic effects on the vascular wall [92]. In addition, adiponectin decreases smooth muscle cell proliferation and TNF*α* expression in macrophages [91].

### 4.3. Chronic Kidney Diseases

Chronic kidney diseases (CKD) and various functional/structural lesions of the kidney (glomerulomegaly, glomerulosclerosis, diabetic nephropathy, carcinoma of the kidney, and nephrolithiasis) are correlated to obesity. The factors that are linked to the development of obesity include, among the others, energy intake and hyperinsulinemia; furthermore, adipokines as leptin and proinflammatory cytokines, as well as adiponectin, may contribute to renal injury [93]. Endothelial dysfunction has been described as the main pathogenic mechanism responsible for CKD while weight loss and restoration of adipokine levels represent crucial factors to ameliorate the progression of renal diseases [94]. In patients with nephrotic syndrome, adiponectin has been found strongly increased, and a direct association with proteinuria has been found [95, 96]. Moreover, a prognostic implication of adiponectin has been hypothesized since hyperadiponectinemia has been associated with mortality in patients with CKD. However, the mechanism by which adiponectin increases in renal failure has not been clarified and the clinical significance of plasma adiponectin level in patients with moderate renal dysfunction is controversial:* in vitro* studies indicated that adiponectin binds to cystatin C, an inhibitor of the cathepsin family, which abrogates, in a dose-dependent manner, the suppressive effects of adiponectin on adhesion molecules induced by TNF-*α*-induced [97]. Moreover, in adiponectin knockout mice, Sharma et al. evidenced high levels of microalbuminuria, oxidative stress, and podocyte damage that are reduced after exogenous adiponectin administration [98].

### 4.4. Atherosclerosis

Multiple mechanisms link obesity with CVDs [82, 99]. Many adipokines mediate the cross talk between adipose tissues, heart, and vasculature in the “adipo-cardiovascular axis”; the altered release of adipokines promotes a prothrombotic state contributing to CVDs and atherosclerosis [19, 100]. Several studies indicate that adiponectin has a beneficial role in CVDs and atherosclerosis. Low serum adiponectin levels are predictors of atherosclerosis and myocardial infarction. In addition, there is a robust association between hypoadiponectinemia and coronary heart disease: clinical trials have confirmed that low levels of adiponectin are associated with higher incidence of cardiovascular events and worse outcome [82]. Interestingly, several studies suggested that the HMW adiponectin is a more accurate independent risk factor for CVDs than total adiponectin level [57, 83]. Serum adiponectin levels have been found to be inversely correlated to a marker of carotid atherosclerosis (intima thickness). However, Sattar et al. reported no significant relationship between serum adiponectin levels and risk of coronary heart disease and another study showed high adiponectin levels as predictor of adverse outcome in patients with acute coronary syndrome [101]. These contradictory results might arise from confounding factors and different oligomers of adiponectin tested. Mice lacking adiponectin have severe neointimal injured arteries, and adiponectin restores neointimal proliferation; moreover, in cultured smooth muscle cells, adiponectin attenuated DNA synthesis induced by growth factors, heparin-binding epidermal growth factor- (EGF-) like growth factor (HB-EGF), basic fibroblast growth factor, and EGF and cell proliferation and migration induced by HB-EGF. In cultured endothelial cells, adiponectin attenuated HB-EGF expression stimulated by tumor necrosis factor alpha [102].* In vitro* studies have proved that adiponectin strongly inhibits the production of inflammatory cytokines and adhesion molecules in endothelial cells; in addition adiponectin reduces the transformation of macrophage to foam cells, inhibits TNF*α* production, and stimulates the production of the anti-inflammatory IL-10 cytokine [103].

### 4.5. Chronic Obstructive Pulmonary Disease

Chronic obstructive pulmonary disease (COPD) is one of the major causes of morbidity and mortality worldwide; systemic inflammation and extrapulmonary comorbidities contribute to the overall disease severity [104–109]. Obesity in COPD is associated with increased symptoms of dyspnoea, poorer health-related quality of life, increased levels of fatigue, and exercise performance limitations [110, 111]. Low BMI is an independent risk factor for mortality in subjects with COPD, and this association is strongest in subjects with severe disease. In humans, adiponectin serum levels are elevated in COPD patients. It is known that levels of total adiponectin are low in smokers without COPD, while high levels are observed in COPD patients [41, 112, 113]. Different studies showed that total serum levels of adiponectin represent a significant diagnostic and prognostic marker of COPD. The oligomerization pattern of adiponectin is altered in COPD; in particular the higher levels of adiponectin are associated with a specific increase of HMW [41, 114]. Protective anti-inflammatory role of HMW oligomers has been demonstrated both* in vivo* and* in vitro* studies. We have shown that in A549 cells exposed to TNF*α* and/or IL1*β*, adiponectin reduces in dose- and time-dependent manner cytotoxic effects of TNF*α* and IL1*β* improving cell viability and decreasing apoptosis [60]. In addition, adiponectin inhibits NF-*κ*B nuclear transactivation and induces the expression of the anti-inflammatory IL10 cytokine via ERK1/2 and AKT through the specific mediation of AdipoR1. Finally, the mouse model lacking adiponectin spontaneously develops a COPD-like phenotype with extrapulmonary effects, including systemic inflammation, body weight loss, and osteoporosis. A protective role of adiponectin on mice lung through inhibition of alveolar macrophage function and vascular homeostasis regulation has been found by Summer et al. [115].

### 4.6. Obstructive Sleep Apnoea Syndrome

Obstructive sleep apnoea syndrome (OSAS) is a highly prevalent condition characterized by repeated disruptions of breathing during sleep [116]. Obesity is a major risk factor for OSA [116]. Systemic inflammation and oxidative stress are thought to play key roles in the activation of a variety of pathological mechanisms that are involved in OSA, including increased cardiovascular sympathetic tone, impaired regulation of coagulation, impaired glucose metabolism, and endothelial dysfunction. Adiponectin levels are significantly lower in patients with OSAS being related to its severity and arterial oxygen saturation. Hargens et al. confirmed that adiponectin levels are lower in the OSAS patients compared to controls [117]. However, two recent works indicate that adiponectin levels are not affected in OSAS. The lack of clarity regarding the role of adiponectin in OSAS is certainly due to the potential coexistence of confounding factors, such as visceral obesity or IR. It is argued that the decrease of adiponectin in OSAS is due to the intermittent hypoxia which causes a decrease in the secretion of total and HMW adiponectin by adipocytes [118, 119].

### 4.7. Diabetic Retinopathy

Obesity is a risk factor for diabetic microvascular complications. In fact, the glucose levels in T2DM are responsible for the increased risk of both microvascular (retinopathy, nephropathy, and neuropathy) and macrovascular complications (ischaemic heart disease, stroke, and peripheral vascular disease) [120]. Diabetic retinopathy, the most frequent diabetic microvascular complication, affects 30–50% of all diabetic patients [120]. Circulating levels of adiponectin decrease both in obesity and in T2DM. Moreover, T2DM patients, suffering from diabetic retinopathy (proliferative as well as nonproliferative), show lower levels of adiponectin than matched patients without retinopathy [121]. Additionally, hypoadiponectinemia is positively correlated with the severity of retinopathy in T2DM [122]. Recently, Costagliola et al. analyzed the levels of vascular endothelial growth factor (VEGF) and adiponectin in the aqueous humor of patients with diabetic proliferative retinopathy (PDR) and found that they were significantly higher than those recorded in control subjects [123, 124]. A possible explanation of this finding may be attributed to the increased blood retinal barrier permeability documented in PDR patients [125]. Another possible explanation could be the local reparative response to endothelial dysfunction; in fact, adiponectin induces endothelial nitric oxide production* in vitro* [126]. Moreover, intravitreal bevacizumab, an angiogenesis inhibitor, significantly reduced the levels of VEGF and adiponectin [123]. The treatment with anti-VEGF agents as bevacizumab significantly reduced macular edema and it is possible that bevacizumab also modulates mediators involved in the pathogenesis of macular edema as adiponectin. This finding could be due to the effect of VEGF inhibition on adipocytes differentiation. In fact,* in vivo* the inhibition of VEGF receptor affects adipocytes differentiation, with a consequent decrease of adipokines secretion. Thus, it is not surprising that the bevacizumab treatment, through a VEGF inhibition, could be responsible for the reduction of adiponectin levels [127].

### 4.8. Cancer

Obesity is a risk factor for many cancers [128]. The cross talk between macrophages, adipocytes, and epithelial cells via obesity-associated hormones may enhance cancer risk and/or progression.

Reports from the International Agency for Research into Cancer and the World Cancer Research Fund (WCRF) show that strong evidence exists for an association of obesity with endometrial, esophageal adenocarcinoma, colorectal, postmenopausal breast, prostate, and renal cancers whereas the less common malignancies are leukemia, non-Hodgkin's lymphoma, multiple myeloma, malignant melanoma, and thyroid tumors [85, 128, 129]. Several obesity-related host factors can influence breast tumor initiation, progression, and/or response to therapy including insulin, insulin-like growth factor-1, leptin, adiponectin, steroid hormones, cytokines, vascular regulators, and inflammation-related molecules [130]. In several human studies, adiponectin has been found to be associated with a number of cancer types: decreased in breast and endometrial cancer but increased in nonsmall cell lung cancer, prostate, gastric, liver, pancreatic, and hematological cancers, colon cancer, and renal cell carcinoma [131–134].* In vitro* studies suggested that in certain cancers, such as colorectal, breast, and liver cancers, adiponectin promotes tumor growth, while in others it suppresses it [64]. Moreover, the intracellular pathway underling adiponectin actions has been investigated. It is well established that adiponectin is able to activate several intracellular pathways including AMPK, MAPK, and PI3K/AKT. AMPK interferes with cellular growth signaling through mTOR, thus inhibiting the promotion of carcinogenesis. On the other hand, adiponectin activates AMPK in several cell lines promoting growth arrest and apoptosis via increased p53 and p21 expression. Furthermore, growth factors activate PI3K which results in the phosphorylation of AKT that promotes cellular growth and proliferation. Adiponectin treatment of breast and colorectal cancer cell lines decreases the phosphorylation of PI3K and AKT while the phosphorylation is increased in lung and pancreas cell lines, leading in both cases to a suppression of tumor growth [55, 60, 68]. The superfamily of MAPKs involves c-Jun N-terminal kinases (JNK) and p38 and extracellular signal-regulated kinases (ERK1/2). ERK1/2 is mitogenic, stimulating cell growth. Adiponectin treatment on hepatocellular carcinoma cell line resulted in increased JNK activation while on a lung cell line in ERK1/2 activation and subsequent apoptosis and suppression of cell growth. On the contrary, endometrial and breast cancer cell lines showed that adiponectin inhibited ERK1/2 signaling, resulting in decreased cellular viability [41]. Increased or decreased expression of AdipoRs has been reported in several cell lines and* in vivo* cancer tissues [41, 135–137]. In addition, AdipoRs expression has been implicated as a prognostic marker for some cancer types since a differential expression was found according to disease stage [135]. Further studies on the role of adiponectin in cancer may facilitate the development of new therapeutic targets.

## 5. Obesity Treatments 

Advances have been made towards the care and prevention of obesity but medical treatments are often unsuccessful even in compliant patients. Today, available treatments include combination of diet, physical exercise, and pharmaceutical regimens where monoagent therapy is not as effective as combination ones. Bariatric surgery is considered the most efficient treatment in severely obese patients (BMI ≥ 40 kg/m^2^) [138, 139]. Adiponectin, showing antihyperglycemic, antiatherogenic, and anti-inflammatory properties, could have important clinical benefits such us enabling the development of therapies for the prevention and/or for treatment of obesity and its obesity-related diseases.

Various therapeutic approaches are targeted to increase adiponectin expression or its activity with different strategies: (1) caloric restriction and physical exercise, (2) administration of inducers, (3) administration of recombinant adiponectin, and (4) peptide mimetic approaches. Many of these interventions have demonstrated therapeutic benefits in animal models of metabolic diseases (Table 1). 

*(1) Caloric Restriction and Physical Exercise.* Caloric restriction increases adiponectin gene expression in humans and animals. In fact, reduction of ~10 to 20% weight in obese subjects significantly increased the expression of adiponectin in WAT and in serum [140]. A statistically relevant increase in adiponectin levels has also been found in response to specific diet regimens: low-fat diet, daily supplementation of fish or omega 3, and fiber supplementation [162–166]. However, adiponectin levels, in most studies, are not modified by regular exercise without diet restriction [167, 168]. Current evidence indicates a possible synergistic effect of physical activity and calorie-restricted diet on adiponectin and its oligomers modulation [143]. However, exercise without significant weight loss does not appear to improve adiponectin levels [142, 143]. In conclusion, dietary management can be an effective therapeutic mean of increasing adiponectin levels. Similar to humans, caloric restriction increases adiponectin gene expression and circulating levels in animal models [169].

*(2) Administration of Inducers.* Drugs like rosiglitazone and pioglitazone, belonging to the thiazolidinedione (TZD) class of PPAR*γ* agonists, have been clinically and experimentally proven to be potent inducers of adiponectin expression [153–155]; accordingly, many of the metabolic effects of rosiglitazone or pioglitazone are absent and/or decreased in adiponectin knockout mice. Recently, statins have been reported to increase circulating adiponectin levels [152]. Metformin (MET) improves peripheral insulin sensitivity and increases insulin mediated skeletal muscle glucose uptake. However, there are studies showing that MET has no effect on intracellular levels and secretion of adiponectin. Further studies will be needed to address these controversies [151]. Several new synthetic drugs such as telmisartan, bezafibrate, rimonabant, and natural products such as anthocyanin, xanthohumol, catechins, and sulfatide have been reported to enhance adiponectin production [146–150]. Recently, curcumin, the components of ginger and red pepper, [6] gingerol, and capsaicin have been identified as stimulators of adiponectin production in mouse and human adipocytes and lowered blood glucose and triglyceride levels in mice [145].

*(3) Administration of Recombinant Adiponectin.* Recombinant adiponectin supplementation has been tested in different animal studies. Liu et al. analyzed adiponectin administration in adiponectin knockout mice after high-fat diet feeding and found amelioration in metabolic profile (glucose handling, insulin signaling, triglycerides levels, and mitochondrial structure and function) [156]. Moreover, Kondo et al. demonstrated in mice and in pig models that adiponectin protects against ischemia/reperfusion injury through its ability to suppress inflammation, apoptosis, and oxidative stress [157]. However, lack of an easy, cost efficient production of the recombinant full-length adiponectin and its brief circulating half-life represents limits to its therapeutic use. Therefore, many researchers have focused on the production of adiponectin variant containing only the C-terminal globular domain [31, 158]. Ge et al. generated an alternative globular adiponectin consisting of three monomers fused to an Fc fragment; the combined effects of single-chain and Fc fusion improved the serum half-life from less than 2 h to close to 2 weeks [33].

*(4) Peptide Mimetic Approaches.* The difficulty of converting adiponectin into a viable drug has been demonstrated; therefore, these days, AdipoRs activation is showing one of the most promising novel therapeutic approaches for treating obesity-related disorders. Otvos et al. found the first-in-class adiponectin receptor agonist, ADP 355 [159]. Sun et al. recently identified, through a high throughput screening against a natural compounds library, nine AdipoRs agonists [160]. Four of them, matairesinol, arctiin, (-)-arctigenin, and gramine, show high affinity for AdipoR1. Four of these compounds, parthenolide, taxifoliol, deoxyschizandrin, and syringing, show high affinity for AdipoR2 [160]. Recently, Okada-Iwabu et al. identified a small AdipoRs agonist, AdipoRon that,* in vitro*, binds to both AdipoR1 and AdipoR2 with high affinity [161]. AdipoRon improves, in different mice models, IR and glucose tolerance showing very similar effects to adiponectin.

## 6. Conclusions

Obesity is a pandemic condition that leads to health impairment by increasing the risk of developing diseases such as T2DM, MetS, CVDs, respiratory disorders, and several types of cancer. The molecular mechanism underlying the development and establishment of obesity needs to be better understood. At present, it is well recognized that fat accumulation in obesity results in an altered expression of several hormones, growth factors, and adipokines. Adiponectin may act as a protective and safe endocrine/paracrine/autocrine factor to prevent the establishment and/or progression of lethal conditions related to obesity. Currently, obesity therapeutic regimes include diet therapy, exercise, and behavior modifications but are often associated with poor treatment outcome and therefore new molecular therapeutic targets need to be investigated. In this context, expression enhancement of adiponectin and its AdipoRs may represent a useful therapeutic approach against obesity and obesity-related diseases. Further research is needed to better understand the pathophysiological role of adiponectin in obesity and obesity-related disorders and to clarify the potential clinical application in humans.

## Figures and Tables

**Table 1:** Summary of Adiponectin as therapeutic target.

Compound	Category	Main results	Pubblications
	Caloric restriction	Increase of adiponectin serum levels. Decrease of the ratio of TNF*α* to adiponectin.	Salas-Salvadó et al., 2006 [140], Weiss et al., 2006 [141]

	Long-term physical exercise	Small to moderate increase in adiponectin levels. Short-term activity is not determinant.	Simpson and Singh, 2008 [142], Rokling-Andersen et al., 2007 [143]

	Caloric restriction + physical exercise	Increase of adiponectin serum levels. Increase of mRNA of AdipoRs in muscle and adipose tissues, mRNA levels of Adiponectin in adipose tissues and in serum.	Rokling-Andersen et al., 2007 [143], Christiansen et al., 2010 [144]

Curcumin, capsaicin, and [6] gingerol	Adiponectin inducer	Promotion of adiponectin endogenous production.	Yamazaki et al., 2008 [145]

Synthetic and natural products	Adiponectin inducer	Anthocyanin enanches adiponectin secretion. Xanthohumol increases adiponectin levels and attenuates diabetes in mice. Rimonabant significantly elevates adiponectin and reduces waist circumference. Telmisartan increases plasma adiponectin levels. Benzafibrate increases adiponectin levels in adipocytes and in serum of mice. sulfatide increases adiponectin production in adipocytes. Catechins enhance the expression and secretion of adiponectin in dose and time dependent manner in adipocytes.	Tsuda et al., 2004 [146], Nozawa, 2005 [147], Moriuchi et al., 2007 [148], Hiuge et al., 2007 [149], Bruun et al., 2007 [150]

Metformin	Adiponectin inducer	Increase of adiponectin serum levels and reduction of BMI and insulin resistance.	Adamia et al., 2007 [151]

Statins	Adiponectin inducer	Increase of adiponectin levels.	Sahebkar, 2013 [152]

Thiazolidinedione (TZD)	Adiponectin inducer	Increase of AdipoRs receptors in adipocytes and macrophages. Increase of adiponectin secretion from adipocytes and of both serum adiponectin concentration and ratio of HMW/total adiponectin.	Tsuchida et al., 2005 [153]; Phillips et al., 2008 [154]; M. Liu and F. Liu, 2009 [155]

*f*Adiponectin	Recombinant adiponectin	Correction of amino acids metabolism altered by high-fat diet. Protection against injury in pig with myocardial ischemia-reperfusion through suppression of inflammation, apoptosis, and oxidative stress.	Liu et al., 2013 [156], Kondo et al., 2010 [157]

*g*Adiponectin	Recombinant adiponectin	Decrease of plasma free fatty acids levels in mice. Induction of weight reduction in mice on high/fat/sucrose diet. Amelioration of atherosclerosis.	Fruebis et al., 2001 [31], Yamauchi et al., 2003 [158]

*g*Adiponectin fused to Fc fragment	Recombinant adiponectin	Improve of the fasting glucose levels and of the tolerance to glucose in mice.	Ge et al., 2010 [33]

ADP 355	AdipoRs agonist	Suppression of tumor growth in cancer cell lines and mice.	Otvos et al., 2011 [159]

Natural compounds library (9 compounds validated)	AdipoRs agonists	agonist demonstrated *by in vitro* tests.	Sun et al., 2013 [160]

AdipoRon	AdipoRs agonist	Amelioration of diabetes in genetically obese rodents and prolongation of the shortness life span of rodents on high-fat diet.	Okada-Iwabu et al., 2013 [161]
